# Hypertension as a prognostic factor in metastatic renal cell carcinoma treated with tyrosine kinase inhibitors: a systematic review and meta-analysis

**DOI:** 10.1186/s12894-019-0481-5

**Published:** 2019-06-07

**Authors:** Yu Liu, Liang Zhou, Yuntian Chen, Banghua Liao, Donghui Ye, Kunjie Wang, Hong Li

**Affiliations:** Department of Urology, Institute of Urology (Laboratory of Reconstructive Urology), West China Hospital, Sichuan University, No.37 Guo Xue Xiang, Chengdu, Sichuan 610041 People’s Republic of China

**Keywords:** Metastatic renal cell carcinoma, Tyrosine kinase inhibitors, Hypertension, Prognosis, Meta-analysis

## Abstract

**Background:**

Conflicting evidence exists regarding the effect of hypertension on the prognosis of metastatic renal cell carcinoma (mRCC) patients treated with tyrosine kinase inhibitors (TKIs). This study aimed to assess the predictive value of TKIs-induced hypertension in patients with mRCC.

**Methods:**

This study was registered in PROSPERO (CRD42019129593). PubMed, Embase, Web of Science and the Cochrane Library database were searched with terms: “renal cell carcinoma”, “hypertension”, “blood pressure”, “tyrosine kinase inhibitor”, “sunitinib”, “axitinib”, “sorafenib” and “pazopanib” until March 21, 2019. Hazard Ratios (HR) and 95% confidence intervals (CI) for progression-free survival (PFS) or overall survival (OS) were extracted and analyzed with Stata 15.0 software. Heterogeneity was assessed using the I^2^ value. Meta-regression, subgroup analysis and sensitivity analysis were also performed to explore heterogeneity. Publication bias was assessed with funnel plots and precisely assessed by Egger’s and Begg’s tests. The quality of evidence of outcomes was generated according to the Grading of Recommendations Assessment, Development, and Evaluation (GRADE).

**Results:**

A total of 4661 patients from 22 studies were included in the study. The results showed that the increase of blood pressure was an effective predictor for longer PFS (HR = 0.59, 95% CI: 0.48–0.71, *p* < 0.001; I^2^ = 77.3%) and OS (HR = 0.57, 95% CI: 0.45–0.70, *p* < 0.001; I^2^ = 77.4%) of patients with mRCC. Subgroup analysis revealed that patients receiving sunitinib and pazopanib could have longer PFS and OS.

**Conclusions:**

This study indicated that TKIs-induced hypertension may be a good predictor for better prognosis of patients with mRCC receiving TKIs treatment, especially using sunitinib or pazopanib.

**Electronic supplementary material:**

The online version of this article (10.1186/s12894-019-0481-5) contains supplementary material, which is available to authorized users.

## Background

Renal cell carcinoma (RCC) is the 9th most common cancer in men and 14th most common cancer in women worldwide [1]. Its incidence (3–6/100,000) and mortality (1.2–2.5/100,000) are increasing rapidly, which has a great negative effect on our society [2, 3]. In addition, about 25–30% of patients have evidence of metastasis upon its diagnosis [4]. Now, vascular endothelial growth factor receptor tyrosine kinase inhibitors (TKIs), like sunitinib, pazopanib, sorafenib or axitinib, are the favored medicine for metastatic RCC (mRCC). Several clinical trials showed that response to TKIs was uncertain (objective response rate was 31–67.4%) [5–8], which indicated the existence of wide inter-individual variation and the lack of reliable factors for predicting the outcomes of mRCC patients. Therefore, a big challenge faced by urologists is how to predict the prognosis of mRCC patients receiving TKIs more precisely.

The occurrence of several adverse events (AEs) during TKIs therapy, such as hypertension, hand-foot syndrome or hypothyroidism, were shown to be correlated with the longer median progression-free survival (PFS) and overall survival (OS) of mRCC patients [9]. Hypertension during TKIs therapy was very common, with incidence ranging from 22 to 81% [9, 10]. Recently, a study found that RCC patients with longer median PFS (>5.3 months) demonstrated a significantly higher incidence of high-grade hypertension (a treatment-associated adverse event) than those with shorter PFS [11]. It indicated that TKIs-induced hypertension may be associated with improved prognosis [12, 13]. However, others reported insignificant results [14, 15]. In 2014, a systematic review and meta-analysis reported that the occurrence of hypertension due to sorafenib therapy may be associated with improved prognosis of patients with cancer. However, this study did not specifically focus on the mRCC patients. Thus, the association between TKIs-induced hypertension and prognosis of mRCC is still controversial. In the present study, we attempt to conduct a systematic review and meta-analysis to assess the predictive value of the TKIs-induced hypertension for PFS and OS in patients with mRCC during TKIs therapy.

## Methods

### Data sources and literature search strategy

We conducted this meta-analysis in accordance with the Preferred Reporting Items for Systematic Reviews and Meta-Analysis statement. This study was registered in PROSPERO (CRD42019129593). A literature search was performed in the following databases: PubMed, Embase, Web of Science and the Cochrane Library database. The latest search was performed on March 21, 2019. The search keywords were (renal cell carcinoma) AND [(tyrosine kinase inhibitor) OR sunitinib OR axitinib OR sorafenib OR pazopanib] AND [hypertension OR (blood pressure). The resulted literatures were further scanned by Endnote X7 (Thomson Corporation, Canada) to exclude duplications followed by title and abstract screening. In addition, we manually searched the references of the literatures for additional eligible studies.

### Inclusion and exclusion criteria

Studies were included in our meta-analysis if the following criteria were met: 1) mRCC patients were treated with TKIs; 2) studies compared Hazard Ratios (HR) between patients with or without TKIs-induced hypertension for PFS or OS.

Studies were excluded based on the following criteria: 1) reviews, meta-analysis, letters, comments, case reports or conference abstracts; 2) duplicated studies and repeated analysis; 3) studies lacking sufficient data for HR and their 95% confidence intervals (CI); 4) studies included mRCC patients received different therapeutic regimen, such as TKIs or radiotherapy.

### Quality of original studies

We assessed the quality of the 22 included studies using the Newcastle-Ottawa Scale (NOS). The total score was 9 stars. Studies awarded 7–9 stars were rated as high-quality, 5–6 stars as moderate-quality, and < 5 stars as low-quality.

### Data extraction

Eligible studies were read thoroughly and carefully to extract the following information, including first author, year, region, sample size, male/female ratio, mean age, histology, number of disease sites, prior nephrectomy, Memorial Sloan Kettering Cancer Center (MSKCC) score, Eastern Cooperative Oncology Group performance status (ECOG PS) score, definition of hypertension, type of analysis (univariate or multivariate), study design (prospective or retrospective), type of TKIs. The primary outcome was HR and 95% CI between patients with or without hypertension for PFS and secondary one for OS. The definition of PFS was time from initiation of TKIs therapy to disease progression or death. OS was defined as time from initiation of TKIs therapy to death. If a study provided both univariate and multivariate analysis results, the multivariate analysis would be selected to achieve higher accuracy. Any discrepancies were resolved by discussion with the third reviewer.

### Data synthesis and statistical analysis

Data were extracted and analyzed with Stata 15.0 software (Stata Corporation, College Station, TX, USA). *P* value<0.05 was considered statistically significant. A merged HR greater than 1 indicated a poorer prognosis for mRCC patients. Heterogeneity was assessed using the I^2^. We considered I^2^ > 50% as an indicator of substantial heterogeneity. A random effects model and a fixed effects model were applied for I^2^>50% and I^2^<50%, respectively. Then, to determine which factors may contribute to heterogeneity, univariate and multivariate meta-regression analysis were performed. The possible factors were year, sample size, gender, mean age, country, ECOG PS, MSKCC score, histology, prior nephrectomy, Number of disease sites, type of analysis (univariate, multivariate), study design (retrospective, prospective), type of TKIs. Then, subgroup analysis was performed to investigate whether different sample size could explain the heterogeneity and whether relationship between hypertension and PFS or OS still exist in different TKIs subgroups. Factor with *P* value < 0.05 meant that it may be the source of heterogeneity. We did sensitivity analysis to find if some original studies may mainly contribute to the heterogeneity. Publication bias was assessed with funnel plots and precisely assessed by Egger’s and Begg’s tests.

#### Quality of evidence

The quality of evidence of the predictive effect of TKIs-induced hypertension for the outcomes in mRCC patients was assessed according to the Grading of Recommendations Assessment, Development, and Evaluation (GRADE) [16].

## Results

### Study selection

The searching process is shown in Additional file 1: Figure S1. A total of 982 studies were searched in the database. We excluded 345 duplicated articles. After screening title and abstract, 26 relevant studies were identified. In addition, three relevant studies were obtained from the references and seven articles were excluded due to lack of HR and 95% CI for PFS or OS. Finally, 22 studies were selected for the meta-analysis.

### Study characteristics and quality

The baseline characteristics of these studies were demonstrated in Table 1. All the studies were published between 2011 and 2017. Of them, 3 were prospective and 19were retrospective. The sample size ranged from 28 to 770 patients. The total number of included patients was 4661 and hypertension occurred in 2932 (62.9%). The male/female ratio included in each study ranged from 1.4 to 3.5%, and the median age of the study patients was between 54 years and 66 years. The histology of most RCC is clear cell (61–100%). Most patients had received nephrectomy, cytokine therapy, targeted therapy or radiation therapy. Among the 22 studies providing HR, four reported univariate HR, which did not adjust for the potential confounding factors. The standard of hypertension or BP increasement during TKIs therapy varied between studies, including systolic blood pressure (SBP) ≥140/135 mmHg, diastolic blood pressure (DBP) ≥90/85 mmHg, mean arterial blood pressure (MAP) > 110 mmHg, an increase in BP (SBP, DBP, MAP) > 10/15 mmHg from baseline, or grades of hypertension according to National Cancer Institute Common Terminology Criteria for Adverse Events Version 3.0 or 4.0 [34, 35]. The quality of the studies varied from a NOS score of 6 to 9, most of which were high-quality (Table 2).

**Table 1:** Baseline characteristics of eligible studies in the meta-analyses

Study	Year	Country	Study design	Sample size	Male/ Female ratio	Mean age	Histology (clear cell%)	Survival analysis	Definition of hypertension	Type of analysis	TKIs	Quality Assessment (NOS Score = 9)
Rini (a) [10]	2011	the USA	R	534	2.3	60.6	98%	PFS, OS	SBP ≥ 140 mmHg, DBP ≥ 90 mmHg	multivariate	SUN	7
Szmit [12]	2011	Poland	R	111	3.0	55.9	100%	PFS, OS	BP ≥ 140/90 mmHg	univariate	SUN	9
Bono [17]	2011	Finland	R	64	1.7	64	92%	PFS	BP > 150/100 mmHg OR blood pressure requiring intensifi cation of pre-existing anti-hypertensive medication.	multivariate	SUN	7
Fujita [18]	2012	Japan	R	41	2.7	64	100%	PFS	–	univariate	SUN	7
Eechoute [19]	2012	Netherlands	R	158	1.7	60	87%	PFS, OS	SBP > 140 mmHg, DBP > 90 mmHg, MAP > 110 mmHg	multivariate	SUN	7
Rini (b) [13]	2013	the USA	R	168	2.5	60	–	PFS, OS	DBP ≥90 mmHg	multivariate	AXI	7
Motzer (a) [20]	2013	the USA	P	350	2.8	61	100%	PFS, OS	SBP > 140 mmHg, DBP > 90 mmHg	multivariate	AXI	6
Motzer (b) [20]	2013	the USA	P	336	2.5	61	100%	PFS, OS	SBP > 140 mmHg, DBP > 90 mmHg	multivariate	SOR	6
Hong [21]	2013	China	R	136	2.0	56	93%	OS	Hypertension class III/IV	multivariate	SUN	7
Nakano [22]	2013	Japan	R	36	3.5	65.8	61%	PFS	grade 1–3 (NCI-CTCAE, version 3.0)	multivariate	SOR	7
Fujita [23]	2014	Japan	R	44	2.7	63.5	95%	PFS	–	multivariate	SUN	7
Eto [24]	2014	Japan	R	64	2.2	63	97%	OS	DBP ≥90 mmHg	–	AXI	7
Rini (c) [25]	2015	the USA	P	203	2.0	61.9	–	PFS	DBP change from baseline ≥10/15 mmHg	–	AXI	7
Zhang (a) [26]	2015	China	R	256	2.5	58	79%	OS	–	multivariate	SOR	7
Kucharz [27]	2015	Poland	R	28	2.1	65	–	PFS	office SBP ≥140 and/or DBP ≥90 mmHg; home SBP ≥135 and/or DPB ≥85 mmHg; pre-existing medication-controlled arterial hypertension and required additional antihypertensive medication during treatment	multivariate	SUN	7
Izzedine [28]	2015	France	R	212	3.4	57.7	86%	PFS, OS	–	multivariate	SUN	8
Donskov [29]	2015	the USA	R	770	2.6	60	98%	PFS	SBP ≥ 140 mmHg	multivariate	SUN	7
Zhang (b) [30]	2016	China	R	134	2.4	59.8	77%	OS	–	multivariate	SOR	7
Goldstein (a) [14]	2016	Australia	R	479	2.2	59.5	–	PFS, OS	MAP change from baseline>10 mmHg	univariate	PAZ	9
Goldstein (b) [14]	2016	Australia	R	506	2.6187	61	–	PFS, OS	SBP > 140 mm H, DBP > 90 mmHg, MAP change from baseline>10 mmHg, SBP change from baseline>10 mmHg	univariate	PAZ	9
Goldstein (c) [14]	2016	Australia	R	475	3.3394	60.9	–	PFS, OS	SBP > 140 mm H, DBP > 90 mmHg, MAP change from baseline>10 mmHg, SBP change from baseline>10 mmHg	univariate	SUN	9
Cecere [31]	2016	Italy	R	38	1.375	61	84.2%	OS	grade ≥ 3 (NCI-CTCAE, version 4.0)	multivariate	PAZ	7
Miyake [32]	2016	Japan	R	50	4.0000	64	80%	PFS	SBP ≥ 140 or DBP ≥ 90 mmHg	multivariate	SUN	7
Fukuda [15]	2016	Japan	R	62	2.4444	66	92%	PFS, OS	–	univariate	SUN	7
Matias [33]	2017	France	P	106	2.3125	54	90%	PFS, OS	grade ≥ 3 (NCI-CTCAE, version 4.0)	univariate	AXI	7

*R* Retrospective, *P* Prospective, *PFS* Progression-free survival, *OS* Overall survival, *SBP* Systolic blood pressure, *DBP* Diastolic blood pressure, MAP ≈ 2/3 DBP + 1/3 SBP; *SUN* Sunitinib, *AXI* Axitinib, *SOR* Sorafenib, *PAZ* Pazopanib, *NCI-CTCAE* National Cancer Institute Common Terminology Criteria for Adverse Events

—: The data were not available in this study

**Table 2:** Newcastle-Ottawa scale score of the reviewed studies

Study	Selection (4 stars)				Comparability (2 stars)	Outcome (3 stars)			Total score
	Representativeness of the hypertensive cohort	Selection of the non-hypertensive cohort	Ascertainment of hypertension	Demonstration that outcome of interest was not present at start of study		Assessment of outcome	Was follow up long enough for outcomes to occur?	Adequacy of follow up of cohort	
Rini (a)	★	★	★	★	–	★	★	★	7
Szmit	★	★	★	★	★★	★	★	★	9
Bono	★	★	★	★	–	★	★	★	7
Fujita	★	★	★	★	–	★	★	★	7
Eechoute	★	★	★	★	–	★	★	★	7
Rini (b)	★	★	★	★	–	★	★	★	7
Motzer	–	★	★	★	–	★	★	★	6
Hong	★	★	★	★	–	★	★	★	7
Nakano	★	★	★	★	–	★	★	★	7
Fujita	★	★	★	★	–	★	★	★	7
Eto	★	★	★	★	–	★	★	★	7
Rini (c)	★	★	★	★	–	★	★	★	7
Zhang (a)	★	★	★	★	–	★	★	★	7
Kucharz	★	★	★	★	–	★	★	★	7
Izzedine	★	★	★	★	★	★	★	★	8
Donskov	★	★	★	★	–	★	★	★	7
Zhang (b)	★	★	★	★	–	★	★	★	7
Goldstein	★	★	★	★	★★	★	★	★	9
Miyake	★	★	★	★	–	★	★	★	7
Fukuda	★	★	★	★	–	★	★	★	7
Matias	★	★	★	★	–	★	★	★	7

—: The data were not available in this study

### Relationship between TKIs-induced hypertension and PFS or OS of mRCC patients

**Fig. 1:**
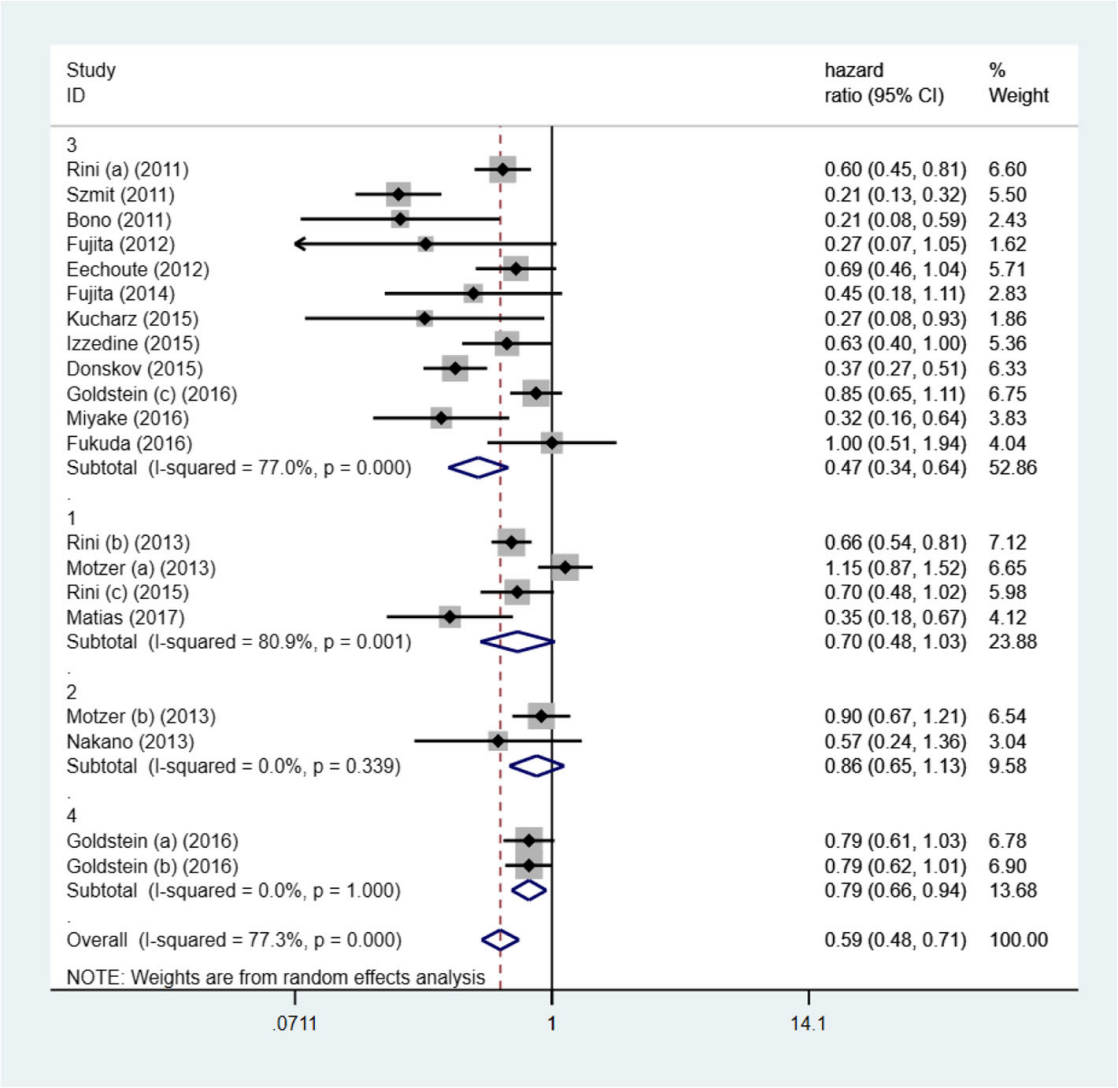
Forest plot reflects the association between TKIs -induced hypertension and oncologic outcomes (progression free survival) in different TKIs subgroups (1: axitinib; 2: sorafenib; 3: sunitinib; 4: pazopanib)

**Fig. 2:**
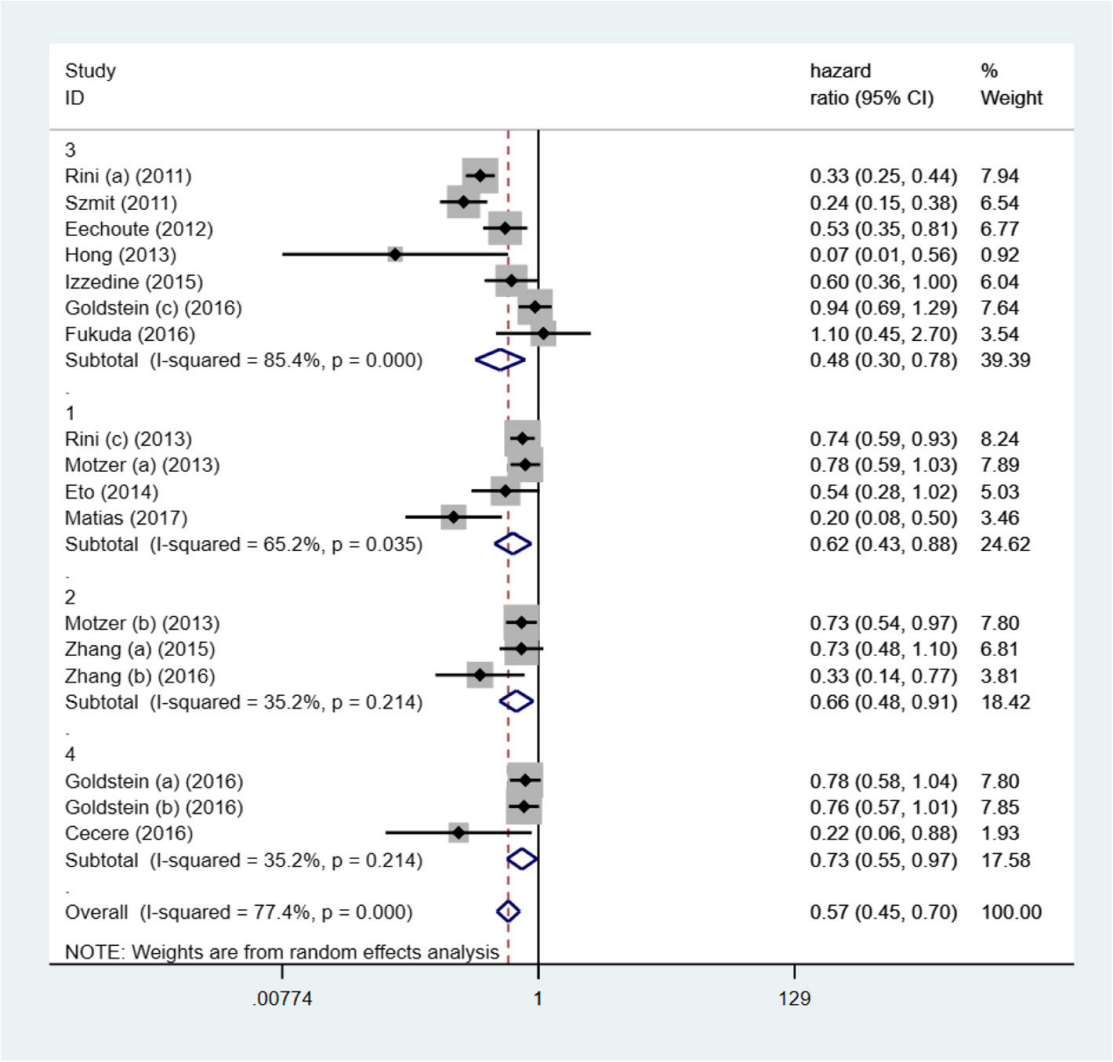
Forest plot reflects the association between TKIs-induced hypertension and oncologic outcomes (overall survival) in different TKIs subgroups (1: axitinib; 2: sorafenib; 3: sunitinib; 4: pazopanib)

HR of PFS and OS were quantitatively synthesized and data were shown in Figs. 1 and 2. Elevated blood pressure was positively correlated with better PFS (HR = 0.59, 95% CI: 0.48–0.71, *p* < 0.001; I^2^ = 77.3%) and OS (HR = 0.57, 95% CI: 0.45–0.70, *p* < 0.001; I^2^ = 77.4%). It meant that patients developing hypertension may have a lower mortality risk and live longer without progression of mRCC. The heterogeneity was obvious between studies.

### Meta-regression analysis

Univariate meta-regression analysis was performed for PFS and results were showed in Table 3. Among the variables above mentioned, only sample size (Adjusted *R*^2^ = 27.34%, *P* = 0.019) might contribute to the inter-study heterogeneity, while others did not (*P* = 0.052–0.942).

**Table 3:** Meta-regression and subgroup analyses of pooled hazard ratios for progression-free survival

Subgroup	Meta-regression							Pooled HR of PFS		Heterogeneity	
	No. of studies	Coefficient	Standard error	T value	*P* value	Tau^2^	Adjusted R^2^	HR (95% CI)	P value	I^2^	*P* value
Year	20	0.060	0.228	0.26	0.795	0.182	−8.86%				
2011–2014								0.56 (0.40–0.77)	<0.001	83.00%	<0.001
2015–2017								0.61 (0.48–0.77)	<0.001	73.60%	<0.001
Sample size	20	0.507	0.197	2.57	0.019	0.122	27.34%				
<200								0.43 (0.30–0.61)	<0.001	72.10%	<0.001
≥200								0.73 (0.60–0.89)	0.002	75%	<0.001
Gender (male/female ratio)	20	−0.117	0.228	−0.51	0.614	0.177	−5.64%				
<2.5								0.66 (0.53–0.82)	<0.001	54.50%	0.025
≥2.5								0.55 (0.40–0.74)	<0.001	84.70%	<0.001
Mean age	20	0.320	0.264	2.21	0.241	0.156	6.69%				
<60								0.44 (0.23–0.86)	0.015	89.40%	<0.001
≥60								0.64 (0.53–0.78)	<0.001	69.70%	<0.001
Country	20	0.168	0.301	0.56	0.584	0.173	−3.31%				
the USA, Europe								0.60 (0.49–0.74)	<0.001	81.40%	<0.001
Asia								0.51 (0.31–0.83)	0.006	39.50%	0.158
ECOG PS (grade 0%)	20	0.036	0.133	0.27	0.79	0.182	−8.59%				
<0.5								0.35 (0.14–0.89)	0.028	87.20%	<0.001
≥0.5								0.64 (0.52–0.79)	<0.001	71.90%	<0.001
MSKCC score (favorable%)	20	0.231	0.111	2.08	0.052	0.123	26.74%				
<0.25								0.43 (0.21–0.87)	0.019	76%	0.002
≥0.25								0.86 (0.73–1.02)	0.076	40.30%	0.137
Histology (clear cell%)	20	−0.139	0.126	−1.11	0.283	0.166	1.11%				
<0.9								0.53 (0.39–0.71)	<0.001	29.30%	0.226
≥0.9								0.51 (0.33–0.79)	0.002	87.90%	<0.001
Prior nephrectomy (%)	20	−0.162	0.129	−1.25	0.226	0.166	1.16%				
<0.9								0.52 (0.40–0.67)	0.296	17.50%	<0.001
≥0.9								0.52 (0.33–0.81)	<0.001	89.30%	0.005
No. of disease sites (1%)	20	−0.253	0.139	−1.81	0.086	0.144	13.81%				
<0.2								0.41 (0.28–0.60)	<0.001	62.40%	0.047
≥0.2								0.52 (0.37–0.74)	<0.001	0	0.594
Type of analysis	20	−0.030	0.231	−0.13	0.898	0.182	−8.91%				
Univariate								0.59 (0.42–0.82)	0.002	82.70%	<0.001
Multivariate								0.58 (0.46–0.75)	<0.001	74.50%	<0.001
Study design	20	0.343	0.257	1.34	0.198	0.157	6.32%				
Retrospective								0.54 (0.44–0.67)	<0.001	74.90%	<0.001
Prospective								0.77 (0.53–1.12)	0.175	76.20%	0.006
Type of TKIs	20	−0.073	0.114	−0.64	0.942	0.178	−10.49%				
Axitinib								0.70 (0.48–1.03)	0.07	80.90%	0.001
Sorafenib								0.86 (0.65–1.13)	0.277	0	0.339
Sunitinib								0.47 (0.34–0.64)	<0.001	77.90%	<0.001
Pazopanib								0.79 (0.66–0.94)	0.010	0	1.000

*No.* number, *HR* hazard ratio, *PFS* progression-free survival, *ECOG PS* Eastern Cooperative Oncology Group performance status, *MSKCC score* Memorial Sloan Kettering Cancer Center score

Multivariate meta-regression analysis revealed that *P* value of sample size changed to 0.025. The overall adjusted R^2^ was 74.84% (*P* = 0.239), which meant that all these factors together could account for 74.84% of heterogeneity.

### Subgroup analysis

Subgroup analysis for PFS revealed that sample size could not change the subgroup heterogeneity significantly (I^2^ = 72.1 and 75%) (Table 3). In addition, for different TKIs, only patients with TKIs-induced hypertension in sunitinib subgroup (HR = 0.47, 95% CI: 0.34–0.64, p<0.001) and pazopanib subgroup (HR = 0.79, 95% CI: 0.66–0.94, *p* = 0.01) could have longer PFS. However, development of hypertension in four different TKIs subgroups could all predict longer OS, including sunitinib subgroup (HR = 0.48, 95% CI: 0.30–0.78, *p* = 0.003), pazopanib subgroup (HR = 0.73, 95% CI: 0.55–0.97, *p* = 0.032), axitinib subgroup (HR = 0.62, 95% CI: 0.43–0.88, *p* = 0.007) and sorafenib subgroup (HR = 0.66, 95% CI: 0.48–0.91, *p* = 0.010) (Figs. 1 and 2).

### Sensitivity analysis

**Fig. 3:**
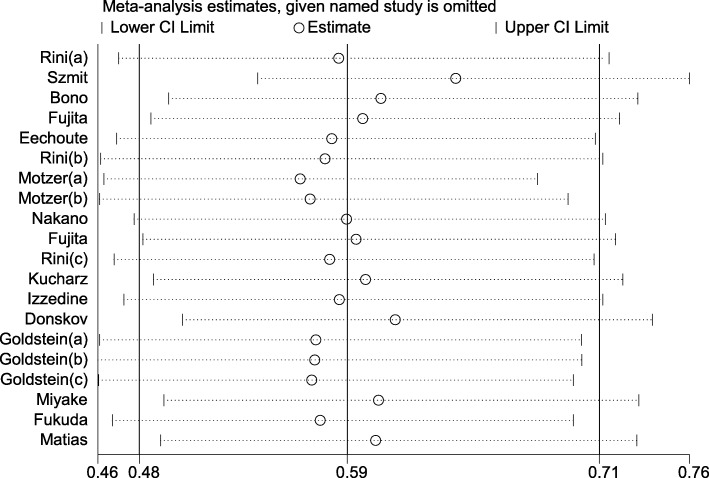
Sensitivity analysis of progression free survival for the evaluation of potential heterogeneity

As shown in Fig. 3, study of “Szmit”, “Motzer (a)” and “Donskov” could affect the heterogeneity for PFS. We excluded these studies and found that I^2^ decreased to 44.6% (*P* = 0.03), with pooled HR = 0.665 (0.579–0.764, *P* = 0.025). Then, we reviewed these studies carefully to find something they had in common. Interestingly, most of the mRCC patients in these three studies had failed one previous cytokine immunotherapy. Maybe it was the reason for high heterogeneity.

### Publication bias

**Fig. 4:**
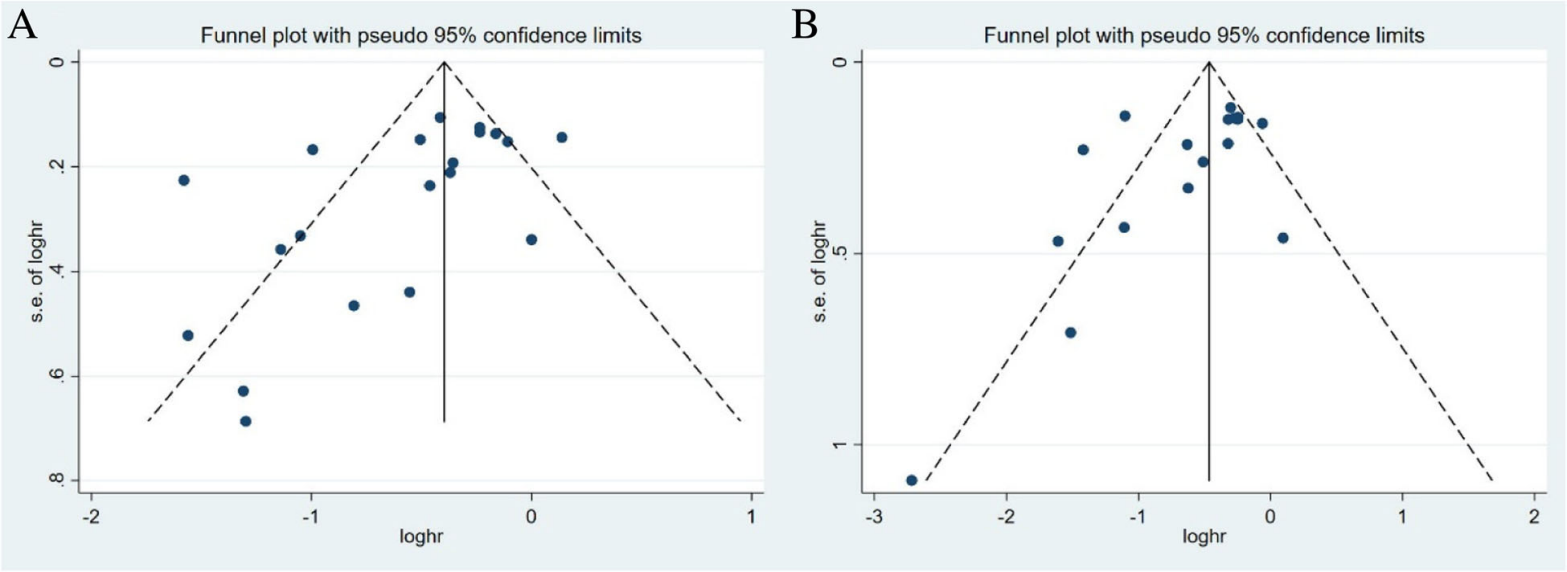
Funnel plot for publication bias. **a** progression free survival, **b** overall survival

We assessed the publication bias with funnel plot and Egger’s and Begg’s tests (Fig. 4). The shape of funnel plots was not symmetric. The Egger’s and Begg’s tests were further performed. The results indicated significant publication bias for studies, with merged PFS (Begg’s test, *P* = 0.015; Egger’s test, *P* = 0.028) and merged OS (Begg’s test, *P* = 0.026; Egger’s test, *P* = 0.085).

### Evaluation of the quality of evidence according to GRADE system

The assessment of the quality of evidence was performed for PFS and OS which were critical in evaluating the outcome of mRCC patients. The quality of evidence of PFS and OS was both “very low” due to retrospective studies, publication bias and high heterogeneity (Table 4).

**Table 4:** Evaluation of the quality of evidence according to GRADE system

Quality assessment							No. of patients		Hazard Ratios (95% CI)	Quality	Importance
No of studies	Design	Risk of bias	Inconsistency	Indirectness	Imprecision	Other considerations	TKI-induced hypertension	Control			
Progression-free survival (follow-up median 5.6–43.2 years; measured with: follow-up)											
20	observational studies	no serious risk of bias	serious^1^	no serious indirectness	no serious imprecision	reporting bias^2^	3021	1327	0.59 (0.48–0.71)	Very low	Critical
Overall survival (follow-up median 5.2–61.8 months; measured with: follow-up)											
17	observational studies	no serious risk of bias	serious^1^	no serious indirectness	no serious imprecision	reporting bias^3^	2313	1804	0.57 (0.45–0.70)	Very low	Critical

^1^The heterogeneity of this outcome was obvious between studies

^2^The shape of funnel plots was not symmetric. The Egger’s and Begg’s tests were further performed. The results indicated significant publication bias for studies, with merged PFS (Begg’s test, *P* = 0.015; Egger’s test, *P* = 0.028).

^3^The shape of funnel plots was not symmetric. The Egger’s and Begg’s tests were further performed. The results indicated significant publication bias for studies, with merged OS (Begg’s test, *P* = 0.026; Egger’s test, *P* = 0.085)

## Discussion

This meta-analysis and systematic review investigated whether TKIs-induced hypertension can predict the prognosis of patients suffering from mRCC. AEs, like hypothyroidism, though shown to be a good predictor of PFS or OS, was usually diagnosed later than hypertension [17, 36]. Thus, it would be better if we can predict the prognosis of mRCC patients based on the TKIs-induced hypertension. It will help urologists decide whether patients should continue this TKIs therapy or not, which may help patients get more suitable treatments as soon as possible. Based on 22 original studies, our results showed that the occurrence of hypertension during treatment may predict better survival for mRCC, with longer PFS and OS. Additionally, when it comes to different TKIs, sunitinib or pazopanib therapy were both associated with longer PFS and OS.

The mechanisms of hypertension induced by TKIs are complicated. First, activation of VEGF receptor-2 via phosphoinositide 3-kinase and its downstream serine protein kinase Akt can stimulate endothelium-derived nitric oxide synthase, leading to the production of nitric oxide (NO). Therefore, the inhibition of VEGF receptor might lead to a decrease in NO bioavailability, followed by vasoconstriction and increased blood pressure (BP) [17, 37–39]. Second, plasma vasoconstrictor endothelin-1, a vasoconstrictor produced by the endothelium, also increased in patients or rats receiving sunitinib [40–42]. Third, NO is also involved in the control of renal and glomerular hemodynamics, tubuloglomerular feedback response, release of renin and sympathetic transmitters, tubular ion transport. As a result, reduction of NO can also result in renal water and sodium retention, leading to hypertension [43]. TKIs may also directly cause renal injury and proteinuria, which could play a role in long-lasting hypertension [44]. Finally, hypertension may also result from structural or functional vascular rarefaction [45–47]. VEGF is also crucial in the maintenance of endothelial viability [48, 49]. Therefore, the inhibition of VEGF and PDGF receptors can induce endothelial cell apoptosis, reduction in capillary density, vascular diameter and microvascular flow, and thus increase the blood pressure.

The reason why TKIs-induced hypertension might be a prognostic factor in patients with mRCC is still not quite clear. The anticarcinogenic effect of TKIs relies on the block of VEGF receptor, which may also lead to hypertension as above mentioned. Thus, when TKIs inhibit the progress of mRCC and prolong the PFS or OS of patients, hypertension may occur at the same time. It may be the reason why the rise of blood pressure could indicate a better prognosis in mRCC patients.

Of note, the intention of therapy is not to drive all patients into a hypertensive state. On the contrary, when hypertension occurs, patients should receive antihypertensive therapy as soon as possible. Additionally, despite the PFS and OS of mRCC patients have prolonged because of TKIs therapy, few comparable benefits have been described in terms of the quality of life [50, 51]. An innovative study that compared sunitinib with pazopanib to evaluate patient preferences suggested that the toxicity profile may have an impact on quality of life and the choice of treatment when two comparable options are on offer [52]. In addition, sunitinib-induced hypertension may be associated with cardiotoxicity or reversible posterior leukoencephalopathy [53–55]. Thus, the panel of National Cancer Institute of the USA had several recommendations for mRCC patients received TKIs [56]. First, urologists should recognize the preexisting hypertension prior to therapy or actively monitor BP throughout treatment. Early use of antihypertensive medication is necessary when high blood pressure occurs, and it is critical for maintaining dose intensity and improving a patient’s quality of life simultaneously [56]. If possible, the goal of management is to reduce BP below 140/90 mmHg. It was also suggested that there was no need to reduce the dose of antihypertensive medication because it did not compromise efficacy of TKIs [57]. Some evidence indicated that using Angiotensin receptor blockers (ARBs) or angiotensin-I-converting enzyme inhibitors may even protect against cancer [58–60]. ARBs were shown to induce apoptosis and inhibit the proliferation of RCC cells in vitro [28]. Thus, some randomized prospective studies could be carried out to see if it is better to use more than one drugs with different antihypertensive mechanisms, which may improve the prognosis [12]. Second, doctors should assess the risk of potential cardiovascular complications.

Several underlying limitations of the study should be presented. First, most eligible studies were retrospective, though with high NOS scores. Second, the reciprocal correlation between the hypertension and other AEs should be noted. Patients with more than one adverse event of any grade had longer PFS and OS [61]. Thus, further studies are needed to analyze the relationship between several adverse events and mRCC. Third, obvious inter-study heterogeneity was observed in this meta analysis, which may be due to different therapies mRCC patients had received before, such as cytokine immunotherapy. In addition, a publication bias was detected in this study. The potential reason may be that studies with non-significant results were not published. High heterogeneity and publication bias weakened the quality of evidence, which may be improved by more randomized prospective trials.

However, our analysis also has some advantages. First, we conducted this review with enough data available for extraction by a comprehensive and robust search strategy. Second, we applied a rigorous inclusion/exclusion criterion. Additionally, most of the studies operated multivariable analysis, which could eliminate the co-factors affecting the PFS and OS of mRCC patients.

## Conclusions

In summary, our analysis of currently available clinical evidence suggests that TKIs-induced hypertension may predict longer PFS and OS in patients with mRCC during TKIs therapy, especially using sunitinib or pazopanib.

## Additional file

Additional file 1: **Figure S1.** Flow chart showing literature searching process of meta-analysis. The search keywords are (renal cell carcinoma) AND [(tyrosine kinase inhibitor) OR sunitinib OR axitinib OR sorafenib OR pazopanib] AND [hypertension OR (blood pressure). (DOCX 140 kb)

## Data Availability

The datasets used and/or analysed during the current study available from the corresponding author on reasonable request.

## Abbreviations

AEs: Adverse events
BP: Blood pressure
CI: Confidence intervals
DBP: Diastolic blood pressure
ECOG PS: Eastern Cooperative Oncology Group performance status
GRADE: Grading of Recommendations Assessment, Development and Evaluation
HR: Hazard Ratios
MAP: Mean arterial blood pressure
mRCC: Metastatic renal cell carcinoma
MSKCC: Memorial Sloan Kettering Cancer Center
NO: Nitric oxide
NOS: Newcastle-Ottawa Scale
OS: Overall survival
PFS: Progression-free survival
RCC: Renal cell carcinoma
SBP: Systolic blood pressure
TKIs: Tyrosine kinase inhibitors

## References

[1] Znaor A, Lortet-Tieulent J, Laversanne M, Jemal A, Bray F (2015). International variations and trends in renal cell carcinoma incidence and mortality. Eur Urol.

[2] Wong MCS, Goggins WB, Yip BHK, Fung FDH, Leung C, Fang Y, Wong SYS, Ng CF (2017). Incidence and mortality of kidney cancer: temporal patterns and global trends in 39 countries. Sci Rep.

[3] Rossi SH, Klatte T (2018). Epidemiology and screening for renal. cancer..

[4] Gupta K, Miller JD, Li JZ, Russell MW, Charbonneau C (2008). Epidemiologic and socioeconomic burden of metastatic renal cell carcinoma (mRCC): a literature review. Cancer Treat Rev.

[5] Naito S, Tsukamoto T, Murai M, Fukino K, Akaza H (2011). Overall survival and good tolerability of long-term use of sorafenib after cytokine treatment: final results of a phase II trial of sorafenib in Japanese patients with metastatic renal cell carcinoma. BJU Int.

[6] Tomita Y, Shinohara N, Yuasa T, Fujimoto H, Niwakawa M, Mugiya S, Miki T, Uemura H, Nonomura N, Takahashi M (2010). Overall survival and updated results from a phase II study of sunitinib in Japanese patients with metastatic renal cell carcinoma. Jpn J Clin Oncol.

[7] Motzer RJ, Hutson TE, Tomczak P, Michaelson MD, Bukowski RM, Rixe O, Oudard S, Negrier S, Szczylik C, Kim ST (2007). Sunitinib versus interferon alfa in metastatic renal-cell carcinoma. N Engl J Med.

[8] Rixe O, Bukowski RM, Michaelson MD, Wilding G, Hudes GR, Bolte O, Motzer RJ, Bycott P, Liau KF, Freddo J (2007). Axitinib treatment in patients with cytokine-refractory metastatic renal-cell cancer: a phase II study. Lancet Oncol.

[9] Ravaud A, Schmidinger M (2013). Clinical biomarkers of response in advanced renal cell carcinoma. Ann Oncol.

[10] Rini BI, Cohen DP, Lu DR, Chen I, Hariharan S, Gore ME, Figlin RA, Baum MS, Motzer RJ (2011). Hypertension as a biomarker of efficacy in patients with metastatic renal cell carcinoma treated with sunitinib. J Natl Cancer Inst.

[11] Li Y, Li S, Zhu Y, Liang X, Meng H, Chen J, Zhang D, Guo H, Shi B (2014). Incidence and risk of sorafenib-induced hypertension: a systematic review and meta-analysis. J Clin Hypertens (Greenwich, Conn).

[12] Szmit S, Langiewicz P, Zlnierek J, Nurzynski P, Zaborowska M, Filipiak KJ, Opolski G, Szczylik C (2012). Hypertension as a predictive factor for survival outcomes in patients with metastatic renal cell carcinoma treated with sunitinib after progression on cytokines. Kidney Blood Press Res.

[13] Rini BI, Garrett M, Poland B, Dutcher JP, Rixe O, Wilding G, Stadler WM, Pithavala YK, Kim S, Tarazi J (2013). Axitinib in metastatic renal cell carcinoma: results of a pharmacokinetic and pharmacodynamic analysis. J Clin Pharmacol.

[14] Goldstein D, Rosenberg JE, Figlin RA, Townsend RR, McCann L, Carpenter C, Pandite L (2016). Is change in blood pressure a biomarker of pazopanib and sunitinib efficacy in advanced/metastatic renal cell carcinoma?. Eur J Cancer (Oxford, England : 1990).

[15] Fukuda H, Kondo T, Iida S, Takagi T, Tanabe K (2016). Treatment-related deterioration of renal function is associated with the antitumor efficacy of sunitinib in patients with metastatic renal cell carcinoma. Urol Oncol.

[16] Guyatt G, Oxman AD, Akl EA, Kunz R, Vist G, Brozek J, Norris S, Falck-Ytter Y, Glasziou P, DeBeer H (2011). GRADE guidelines: 1. Introduction-GRADE evidence profiles and summary of findings tables. J Clin Epidemiol.

[17] Bono P, Rautiola J, Utriainen T, Joensuu H (2011). Hypertension as predictor of sunitinib treatment outcome in metastatic renal cell carcinoma. Acta Oncol (Stockholm, Sweden).

[18] Fujita T, Iwamura M, Ishii D, Tabata K, Matsumoto K, Yoshida K, Baba S (2012). C-reactive protein as a prognostic marker for advanced renal cell carcinoma treated with sunitinib. Int J Urol.

[19] Eechoute K, van der Veldt AA, Oosting S, Kappers MH, Wessels JA, Gelderblom H, Guchelaar HJ, Reyners AK, van Herpen CM, Haanen JB (2012). Polymorphisms in endothelial nitric oxide synthase (eNOS) and vascular endothelial growth factor (VEGF) predict sunitinib-induced hypertension. Clin Pharmacol Ther.

[20] Motzer RJ, Escudier B, Tomczak P, Hutson TE, Michaelson MD, Negrier S, Oudard S, Gore ME, Tarazi J, Hariharan S (2013). Axitinib versus sorafenib as second-line treatment for advanced renal cell carcinoma: overall survival analysis and updated results from a randomised phase 3 trial. Lancet Oncol.

[21] Hong YP, Yao XD, Zhu Y, Ye DW, Shi GH, Zhang SL, Dai B, Zhang HL, Shen YJ, Zhu YP (2013). Exploratory analysis of the effect of toxicity of sunitinib on the clinical outcome of patients with advanced renal cell carcinoma. Zhonghua Yi Xue Za Zhi.

[22] Nakano K, Komatsu K, Kubo T, Natsui S, Nukui A, Kurokawa S, Kobayashi M, Morita T (2013). Hand-foot skin reaction is associated with the clinical outcome in patients with metastatic renal cell carcinoma treated with sorafenib. Jpn J Clin Oncol.

[23] Fujita T, Wakatabe Y, Matsumoto K, Tabata K, Yoshida K, Iwamura M (2014). Leukopenia as a biomarker of sunitinib outcome in advanced renal cell carcinoma. Anticancer Res.

[24] Eto M, Uemura H, Tomita Y, Kanayama H, Shinohara N, Kamei Y, Fujii Y, Umeyama Y, Ozono S, Naito S (2014). Overall survival and final efficacy and safety results from a Japanese phase II study of axitinib in cytokine-refractory metastatic renal cell carcinoma. Cancer Sci.

[25] Rini BI, Melichar B, Fishman MN, Oya M, Pithavala YK, Chen Y, Bair AH, Grunwald V (2015). Axitinib dose titration: analyses of exposure, blood pressure and clinical response from a randomized phase II study in metastatic renal cell carcinoma. Ann Oncol.

[26] Zhang HL, Qin XJ, Wang HK, Gu WJ, Ma CG, Shi GH, Zhou LP, Ye DW (2015). Clinicopathological and prognostic factors for long-term survival in Chinese patients with metastatic renal cell carcinoma treated with sorafenib: a single-center retrospective study. Oncotarget.

[27] Kucharz J, Dumnicka P, Kuzniewski M, Kusnierz-Cabala B, Herman RM, Krzemieniecki K (2015). Co-occurring adverse events enable early prediction of progression-free survival in metastatic renal cell carcinoma patients treated with sunitinib: a hypothesis-generating study. Tumori.

[28] Izzedine H, Derosa L, Le Teuff G, Albiges L, Escudier B (2015). Hypertension and angiotensin system inhibitors: impact on outcome in sunitinib-treated patients for metastatic renal cell carcinoma. Ann Oncol.

[29] Donskov F, Michaelson MD, Puzanov I, Davis MP, Bjarnason GA, Motzer RJ, Goldstein D, Lin X, Cohen DP, Wiltshire R (2015). Sunitinib-associated hypertension and neutropenia as efficacy biomarkers in metastatic renal cell carcinoma patients. Br J Cancer.

[30] Zhang Y, Li Y, Cai Y, Wang K, Li H (2016). Efficacy of sorafenib correlates with Memorial Sloan-Kettering Cancer Center (MSKCC) risk classification and bone metastasis in Chinese patients with metastatic renal cell carcinoma. Cell Oncol (Dordr).

[31] Cecere SC, Rossetti S, Cavaliere C, Della Pepa C, Di Napoli M, Crispo A, Iovane G, Piscitelli R, Sorrentino D, Ciliberto G (2016). Pazopanib in metastatic renal Cancer: a “real-world” experience at National Cancer Institute “Fondazione G. Pascale”. Front Pharmacol.

[32] Miyake M, Kuwada M, Hori S, Morizawa Y, Tatsumi Y, Anai S, Hosokawa Y, Hayashi Y, Tomioka A, Otani T (2016). The best objective response of target lesions and the incidence of treatment-related hypertension are associated with the survival of patients with metastatic renal cell carcinoma treated with sunitinib: a Japanese retrospective study. BMC Res Notes.

[33] Matias M, Le Teuff G, Albiges L, Guida A, Brard C, Bacciarelo G, Loriot Y, Massard C, Lassau N, Fizazi K (2017). Real world prospective experience of axitinib in metastatic renal cell carcinoma in a large comprehensive cancer Centre. Eur J Cancer (Oxford, England: 1990).

[34] Trotti A, Colevas AD, Setser A, Rusch V, Jaques D, Budach V, Langer C, Murphy B, Cumberlin R, Coleman CN (2003). CTCAE v3.0: development of a comprehensive grading system for the adverse effects of cancer treatment. Semin Radiat Oncol.

[35] Chen AP, Setser A, Anadkat MJ, Cotliar J, Olsen EA, Garden BC, Lacouture ME (2012). Grading dermatologic adverse events of cancer treatments: the common terminology criteria for adverse events version 4.0. J Am Acad Dermatol.

[36] Buda-Nowak A, Kucharz J, Dumnicka P, Kuzniewski M, Herman RM, Zygulska AL, Kusnierz-Cabala B (2017). Sunitinib-induced hypothyroidism predicts progression-free survival in metastatic renal cell carcinoma patients. Med Oncol (Northwood, London, England).

[37] Aparicio-Gallego G, Blanco M, Figueroa A, Garcia-Campelo R, Valladares-Ayerbes M, Grande-Pulido E, Anton-Aparicio L (2011). New insights into molecular mechanisms of sunitinib-associated side effects. Mol Cancer Ther.

[38] Hayman SR, Leung N, Grande JP, Garovic VD (2012). VEGF inhibition, hypertension, and renal toxicity. Curr Oncol Rep.

[39] Robinson ES, Khankin EV, Karumanchi SA, Humphreys BD (2010). Hypertension induced by vascular endothelial growth factor signaling pathway inhibition: mechanisms and potential use as a biomarker. Semin Nephrol.

[40] Merkus D, Sorop O, Houweling B, Boomsma F, van den Meiracker AH, Duncker DJ (2006). NO and prostanoids blunt endothelin-mediated coronary vasoconstrictor influence in exercising swine. Am J Phys Heart Circ Phys.

[41] Kappers MH, van Esch JH, Sluiter W, Sleijfer S, Danser AH, van den Meiracker AH (2010). Hypertension induced by the tyrosine kinase inhibitor sunitinib is associated with increased circulating endothelin-1 levels. Hypertension (Dallas, Tex : 1979).

[42] Kappers MH, Smedts FM, Horn T, van Esch JH, Sleijfer S, Leijten F, Wesseling S, Strevens H, Jan Danser AH, van den Meiracker AH (2011). The vascular endothelial growth factor receptor inhibitor sunitinib causes a preeclampsia-like syndrome with activation of the endothelin system. Hypertension (Dallas, Tex : 1979).

[43] Zou AP, Cowley AW (1999). Role of nitric oxide in the control of renal function and salt sensitivity. Curr Hypertens Rep.

[44] Khan G, Golshayan A, Elson P, Wood L, Garcia J, Bukowski R, Rini B (2010). Sunitinib and sorafenib in metastatic renal cell carcinoma patients with renal insufficiency. Ann Oncol.

[45] Lacouture ME, Reilly LM, Gerami P, Guitart J (2008). Hand foot skin reaction in cancer patients treated with the multikinase inhibitors sorafenib and sunitinib. Ann Oncol.

[46] Steeghs N, Gelderblom H, Roodt JO, Christensen O, Rajagopalan P, Hovens M, Putter H, Rabelink TJ, de Koning E (2008). Hypertension and rarefaction during treatment with telatinib, a small molecule angiogenesis inhibitor. Clin Cancer Res.

[47] Hubner N, Yagil C, Yagil Y (2006). Novel integrative approaches to the identification of candidate genes in hypertension. Hypertension (Dallas, Tex: 1979).

[48] Rees ML, Khakoo AY (2011). Molecular mechanisms of hypertension and heart failure due to antiangiogenic cancer therapies. Heart Fail Clin.

[49] Lee S, Chen TT, Barber CL, Jordan MC, Murdock J, Desai S, Ferrara N, Nagy A, Roos KP, Iruela-Arispe ML (2007). Autocrine VEGF signaling is required for vascular homeostasis. Cell.

[50] Cella D, Pickard AS, Duh MS, Guerin A, Mishagina N, Antras L, Neary MP, McCann L, Hodge R, Sternberg CN (2012). Health-related quality of life in patients with advanced renal cell carcinoma receiving pazopanib or placebo in a randomised phase III trial. Eur J Cancer (Oxford, England: 1990).

[51] Bukowski R, Cella D, Gondek K, Escudier B (2007). Effects of sorafenib on symptoms and quality of life: results from a large randomized placebo-controlled study in renal cancer. Am J Clin Oncol.

[52] Escudier B, Porta C, Bono P, Powles T, Eisen T, Sternberg CN, Gschwend JE, De Giorgi U, Parikh O, Hawkins R (2014). Randomized, controlled, double-blind, cross-over trial assessing treatment preference for pazopanib versus sunitinib in patients with metastatic renal cell carcinoma: PISCES study. J Clin Oncol.

[53] Chu TF, Rupnick MA, Kerkela R, Dallabrida SM, Zurakowski D, Nguyen L, Woulfe K, Pravda E, Cassiola F, Desai J (2007). Cardiotoxicity associated with tyrosine kinase inhibitor sunitinib. Lancet (London, England).

[54] Kapiteijn E, Brand A, Kroep J, Gelderblom H (2007). Sunitinib induced hypertension, thrombotic microangiopathy and reversible posterior leukencephalopathy syndrome. Ann Oncol.

[55] Padhy BM, Shanmugam SP, Gupta YK, Goyal A (2011). Reversible posterior leucoencephalopathy syndrome in an elderly male on sunitinib therapy. Br J Clin Pharmacol.

[56] Maitland ML, Bakris GL, Black HR, Chen HX, Durand JB, Elliott WJ, Ivy SP, Leier CV, Lindenfeld J, Liu G (2010). Initial assessment, surveillance, and management of blood pressure in patients receiving vascular endothelial growth factor signaling pathway inhibitors. J Natl Cancer Inst.

[57] Langenberg MH, van Herpen CM, De Bono J, Schellens JH, Unger C, Hoekman K, Blum HE, Fiedler W, Drevs J, Le Maulf F (2009). Effective strategies for management of hypertension after vascular endothelial growth factor signaling inhibition therapy: results from a phase II randomized, factorial, double-blind study of Cediranib in patients with advanced solid tumors. J Clin Oncol.

[58] Rao GA, Mann JR, Shoaibi A, Pai SG, Bottai M, Sutton SS, Haddock KS, Bennett CL, Hebert JR (2013). Angiotensin receptor blockers: are they related to lung cancer?. J Hypertens.

[59] Lever AF, Hole DJ, Gillis CR, McCallum IR, McInnes GT, MacKinnon PL, Meredith PA, Murray LS, Reid JL, Robertson JW (1998). Do inhibitors of angiotensin-I-converting enzyme protect against risk of cancer?. Lancet (London, England).

[60] Makar GA, Holmes JH, Yang YX (2014). Angiotensin-converting enzyme inhibitor therapy and colorectal cancer risk. J Natl Cancer Inst.

[61] Iacovelli R, Verri E, Cossu Rocca M, Aurilio G, Cullura D, de Cobelli O, Nole F (2017). Prognostic role of the cumulative toxicity in patients affected by metastatic renal cells carcinoma and treated with first-line tyrosine kinase inhibitors. Anti-Cancer Drugs.

