# Supplementary material for: Hypertension as a prognostic factor in metastatic renal cell carcinoma treated with tyrosine kinase inhibitors: a systematic review and meta-analysis
Source: BMC Urol. 2019 Jun 7;19:49. doi: 10.1186/s12894-019-0481-5 (PMC6555944; doi:10.1186/s12894-019-0481-5)
Supplement: Supplementary file 1 — Figure S1. Flow chart showing literature searching process of meta-analysis. The search keywords are (renal cell carcinoma) AND [(tyrosine kinase inhibitor) OR sunitinib OR axitinib OR sorafenib OR pazopanib] AND [hypertension OR (blood pressure). (DOCX 140 kb) [file 12894_2019_481_MOESM1_ESM.docx]

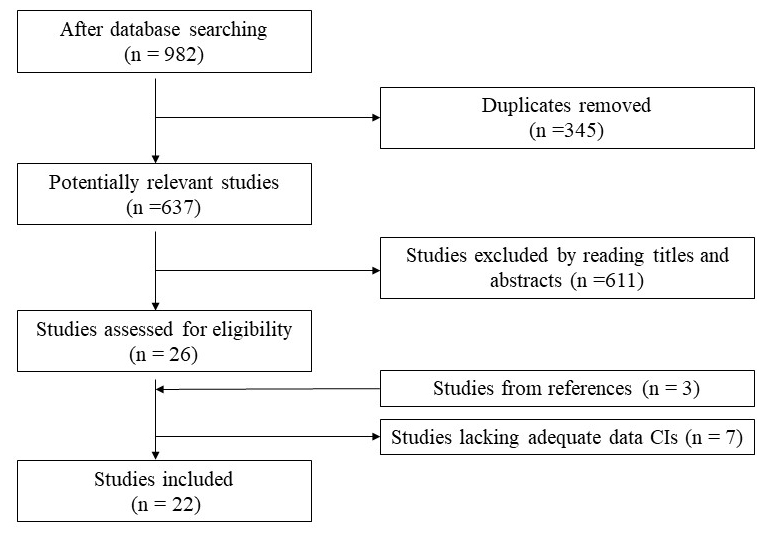


Supplementary Figure 1 Flow chart showing literature searching process of meta-analysis. The search keywords are (renal cell carcinoma) AND [(tyrosine kinase inhibitor) OR sunitinib OR axitinib OR sorafenib OR pazopanib] AND [hypertension OR (blood pressure).
